# Developmental Programming of Hypertension and Kidney Disease

**DOI:** 10.1155/2012/760580

**Published:** 2012-11-28

**Authors:** Euming Chong, Ihor V. Yosypiv

**Affiliations:** ^1^Section of Neonatology, Department of Pediatrics, Hypertension and Renal Center of Excellence, Tulane University Health Sciences Center, New Orleans, LA 70112, USA; ^2^Section of Pediatric Nephrology, Department of Pediatrics, Hypertension and Renal Center of Excellence, Tulane University Health Sciences Center, New Orleans, LA 70112, USA

## Abstract

A growing body of evidence supports the concept that changes in the intrauterine milieu during “sensitive” periods of embryonic development or in infant diet after birth affect the developing individual, resulting in general health alterations later in life. This phenomenon is referred to as “developmental programming” or “developmental origins of health and disease.” The risk of developing late-onset diseases such as hypertension, chronic kidney disease (CKD), obesity or type 2 diabetes is increased in infants born prematurely at <37 weeks of gestation or in low birth weight (LBW) infants weighing <2,500 g at birth. Both genetic and environmental events contribute to the programming of subsequent risks of CKD and hypertension in premature or LBW individuals. A number of observations suggest that susceptibility to subsequent CKD and hypertension in premature or LBW infants is mediated, at least in part, by reduced nephron endowment. The major factors influencing *in utero* environment that are associated with a low final nephron number include uteroplacental insufficiency, maternal low-protein diet, hyperglycemia, vitamin A deficiency, exposure to or interruption of endogenous glucocorticoids, and ethanol exposure. This paper discusses the effect of premature birth, LBW, intrauterine milieu, and infant feeding on the development of hypertension and renal disease in later life as well as examines the role of the kidney in developmental programming of hypertension and CKD.

## 1. Introduction 

Despite the availability of a number of treatment options for hypertension, cardiovascular, and renal disease, the prevalence, morbidity, and mortality of these diseases in children and adults remain very high [1]. Therefore, elucidation of the causality and pathogenesis of these diseases is critical. Studies by Widdowson and McCance in the 1960s demonstrated that acceleration or retardation of the rate of growth induced by malnutrition during early postnatal life in rats led to distinct and different effects on anatomical, physiological, and chemical development [2]. In the 1980s, studies by Barker demonstrated that systolic blood pressure in older children is inversely related to their birth weight [3]. Around the same time, Brenner hypothesized that early loss of nephron mass results in hyperfiltration of remaining nephrons leading to subsequent hypertension, proteinuria, and progressive kidney injury [4]. These and subsequent studies have provided initial evidence that a suboptimal *in utero* environment may predispose or “program” an individual to an increased risk of developing renal or cardiovascular disease in later life [5–9]. Although a number of potential mechanisms underlying developmental origins of disease have been proposed, a likely feature of many of these mechanisms is interruption of normal kidney morphogenesis resulting in a reduced number of nephrons and aberrant development of the kidney vasculature [5]. In this paper, we discuss biological and molecular mechanisms linking pre- and postnatal cues with nephron endowment, vascularization of the developing kidney, and programming of hypertension and renal disease during later life. 

## 2. Major Steps in Morphologic Development of the Kidney

Evidence derived from animal models and human studies has demonstrated that the final number of nephrons can be decreased by adverse prenatal conditions and predispose to later kidney disease [6]. New nephron formation during embryonic kidney development is driven by the branching ureteric bud (UB) that originates from the nephric duct on the 5th week of gestation in humans (embryonic day E10.5 in mice) [10]. Throughout its iterative branching, each UB tip induces nephron progenitors to form nephrons, thus forming the metanephric kidney [5]. UB itself will form the renal collecting ducts, calyces, pelvis, and ureter. The mature human kidney has an average of ~785,000 (range: 210,332–1,825,380) nephrons [11]. As the normal number of nephrons varies widely in humans, a kidney with a decreased complement of nephrons may have less renal reserve to adapt to dietary changes or compensate for renal injury. Vascularization of the metanephros is synchronized with epithelial nephrogenesis [12]. In the mouse kidney, the first arterioles are detected on E15-E16 (mouse gestation is 21 days) [12]. Formation of the metanephros and renal vascularization are directed by multiple gene networks [10, 12]. Aberrant expression or signaling of either the glial cell-derived neurotrophic factor (GDNF)/Ret growth factor/receptor pair, transcription factors Six2 and Pax2, or the renin-angiotensin system components are probably involved in the programming of nephron development [10]. Nephrogenesis continues until 34–36 weeks of fetal life in humans. Following acquisition of the full complement of nephrons between 34 to 36 weeks of gestation in humans, new nephrons cannot be formed, and subsequent glomerular development occurs *via* hypertrophy. Notably, faulty metanephric organogenesis leads to reduced nephron endowment, abnormalities in renal vasculature, renal hypoplasia, hypertension, and congenital anomalies of the kidney and urinary tract (CAKUT), the major cause of renal failure in children [13]. Despite significant progress in our understanding of morphological events and genetic programs that direct nephrogenesis, the underlying molecular mechanisms that account for a decreased final nephron number in response to suboptimal *in utero* or perinatal environment and how alterations in the kidney structure may impact disease risk in later life are incompletely understood. Below, we discuss disparities in nephron endowment in low birth weight (LBW) and premature infants and explore the link of faulty nephrogenesis with susceptibility to CKD and hypertension in later life. 

## 3. Roles of LBW and Prematurity in Kidney Development

### 3.1. Introduction

LBW (birth weight <2500 g) results from either preterm birth (at <37 weeks of gestation) or intrauterine growth restriction (IUGR). The term “IUGR” is used to designate a fetus that has not reached its growth potential because of genetic or environmental factors. The most widely used definition of IUGR is a fetus whose estimated weight is below the 10th percentile for its gestational age (GA). IUGR results in the birth of an infant who is small for gestational age (SGA). In the absence of IUGR, preterm or full-term infants are born appropriate for gestational age (AGA). Importantly, IUGR is a frequent comorbidity of both preterm or term births [14]. 

### 3.2. Impact of LBW on Nephrogenesis, Blood Pressure, and CKD

Animal and human studies have demonstrated the association of LBW with later reduction in glomerular number, renal dysfunction, and hypertension. For example, glomerular number is directly correlated with birth weight, whereas glomerular volume is inversely correlated with birth weight in LBW infants [15]. LBW infants have smaller kidneys and decreased nephron number [16–20]. LBW SGA infants exhibit a 30–35% reduction in nephron number and a higher risk for developing hypertension [21–24]. The observed reduction in nephron endowment is accompanied by endothelial dysfunction that might be secondary to either impaired angiogenesis, decreased production, or function of nitric oxide [23, 24]. In a study of 56 LBW children with IUGR at 4–6 years of age, systolic and diastolic BP were significantly higher compared to the control group [25]. These observations have been confirmed by other studies [26–28]. In a 2012 meta-analysis, LBW was associated with increased odds (1.2) for the development of hypertension [29]. The differences in blood pressure between LBW and normal birth weight individuals are amplified with age [30]. With regard to the correlation between LBW and kidney injury, IUGR is associated with combined odds for microalbuminuria (1.81), ESRD (1.58), and lower GFR (1.79) [31]. A recent meta-analysis demonstrates that the risk for developing proteinuria, decreased kidney function, or ESRD is increased by 81, 79 and 58%, respectively, in LBW neonates [8]. Proteinuria, a reliable predictor of progressive kidney disease, is also more common among adults born as LBW infants [31, 32]. Adults who were born extremely premature and with LBW have an increased incidence of focal segmental glomerulosclerosis (FSGS) with associated proteinuria [33]. These findings are in agreement with Brenner hypothesis that early loss of nephron mass results in hyperfiltration of remaining nephrons leading to secondary FSGS, proteinuria, and progressive kidney injury [4, 34, 35]. Unfortunately, it is not possible to dissociate the effects of prematurity from LBW on the outcomes observed in this study. A systematic review and meta-analysis of observational studies by White et al. examined the association of LBW with the future risk of CKD [9]. This work assessed 31 cohort and case-control studies that included data for birth weight and kidney function at greater than 12 months of age. Significant associations were found between birth weight and low estimated GFR, proteinuria, and ESRD. This analysis identified a consistent association between LBW and subsequent risk of CKD. Animal models have also demonstrated the association of LBW with later hypertension or renal injury. For example, LBW due to ligation of uterine arteries in the rabbit model is associated with reduced number of glomeruli in an offspring [36]. LBW induced by maternal dietary protein restriction during gestation causes a 28-29% decrease in glomerular number and a higher blood pressure at 8 weeks of age in the rat [37]. Restriction of maternal protein intake during rat pregnancy results in LBW, elevated conscious mean arterial pressure, reduced GFR, decreased number of glomeruli, glomerulomegaly and no difference the total volume of all glomeruli in adulthood [38]. Collectively, multiple studies demonstrate that LBW imparts a high risk of low nephron endowment and glomerular hypertrophy. To sustain adequate kidney function following completion of nephrogenesis, remaining glomeruli will need to undergo compensatory hypertrophy, an adaptation that may result in an accelerated loss of functioning nephrons. Resulting decreased filtration surface area may lead to subsequent hypertension *via* limitation in renal sodium excretion. In view of a possible causal relationship between LBW, nephron endowment, hypertension, and CKD, continued assessment of the mechanisms associated with fetal life events are warranted to further define this risk. 

### 3.3. Impact of Prematurity on Nephrogenesis, Blood Pressure, and CKD

More than 12% of infants in the United States are born preterm (at <37 weeks of gestation), illustrating that many neonates enter extrauterine life during active nephrogenesis [49]. Determination of the effect of prematurity *per se* on renal consequences and developmental programming of hypertension and CKD in humans is hampered by the fact that many premature neonates that exhibit IUGR have multiple health problems and are exposed to various medications that may influence nephrogenesis. For example, acute kidney injury (AKI) is observed in 8%–24% of preterm neonates [50]. Use of NSAIDs, aminoglycoside antibiotics, and diuretics have also been shown to potentially hamper nephrogenesis in prematurely born neonates [51]. An autopsy study of premature AGA neonates demonstrated that prematurity alone, without IUGR, is associated with lower radial glomerular counts (RGCs) compared with full-term controls and that glomerular number correlates directly with gestational age [39] (Table 1). RGCs in preterm infants with AKI surviving >40 days were lower than in those without AKI, whereas glomerular surface area was highest in preterm neonates without AKI [39]. Thus, nephrogenesis continues postnatally in preterm neonates and is inhibited by AKI. In addition, preterm birth results in glomerular hypertrophy that may lead to hyperfiltration and subsequent renal injury [39]. Several studies suggest that the presence of IUGR in premature infants does not further increase the risk of poor renal growth or development of subsequent hypertension. In this regard, ultrasonography performed at 20 years of age showed decreased kidney size in individuals born prematurely at <32 weeks of gestation compared with full-term controls and no difference in kidney size between SGA and AGA individuals [40]. Thus, either IUGR had no effect on renal size in premature individuals or the lack of significant differences might be due to insufficient power of this study. In contrast, a study by Drougia et al. reported that kidney length (adjusted for body weight and surface area) is reduced in children who were born premature and SGA compared with those who were premature and AGA [18]. Premature AGA infants have reduced kidney size at birth compared with AGA term neonates and demonstrate no significant improvement in kidney growth with age at 18 months of age [20]. Of note, kidney volume at birth or 18 months of age was lower in premature SGA than premature AGA infants, suggesting that reduced weight for GA has a stronger impact on renal growth than gestational age alone [20]. Prevalence of hypertension at 19 years of age was increased in premature neonates born at <32 weeks of gestation, but this was not apparently related to the extent of IUGR [43]. Similar observations were made by Singhal et al. showing that blood pressure measured at 15 years of age did not differ among full-term, preterm AGA, or preterm SGA (with IUGR) individuals [44]. However, endothelial-dependent vasodilation was reduced in preterm SGA compared with preterm AGA or full-term individuals. These results do not support the hypothesis that prematurity alone leads to elevated blood pressure later in life. In contrast, a large Swedish study reported an inverse association of systolic blood pressure measured at 18 years of age with GA alone or with GA adjusted for birth weight (IUGR), but not with birth length adjusted for GA [45]. The inverse association of GA with blood pressure was larger when adjusted for birth weight, suggesting that IUGR might further increase the risk of subsequent hypertension in premature infants. The authors proposed that the rate of accretion of fetal soft tissue mass rather than of linear bone growth is associated with programming of elevated blood pressure. Kistner et al. reported that systolic ambulatory blood pressure was higher in adult woman born preterm AGA compared with either term AGA or term SGA [46]. Mean blood pressure and brachioradial artery pulse wave velocity, and hence arterial stiffness, were higher in premature SGA compared with premature AGA or term AGA infants when measured at 8 ± 1.7 years of age [47]. The discrepancies in findings among these studies may relate to differences in methodologies used to measure blood pressure. Overall, available evidence indicates that reduced nephron endowment after premature birth does not necessarily result in hypertension later in life. It is conceivable that elevated blood pressure may ensue only when the functional reserve of remaining nephrons is depleted below certain threshold. 

A lower GFR and a higher prevalence of microalbuminuria were observed in SGA compared with AGA premature infants born at <32 weeks of gestation, suggesting that the presence of IUGR might further increase the risk of progressive renal failure and kidney injury in premature infants [31]. In accord with these findings, GFR measured by inulin clearance at mean age of 7.6 years in children was lower in SGA compared with AGA premature individuals [48]. However, urine albumin/creatinine ratio or blood pressure did not differ. Studies in nonhuman primates demonstrate that prematurity without IUGR can cause abnormal kidney development [42]. Preterm baboons delivered at 125 (term = 185) days of gestation were studied after 21 days of extrauterine life and compared to GA-matched controls delivered and studied at 146 (125 + 21) days of gestation. Kidneys of preterm baboons were larger, had decreased glomerular density, enlarged glomeruli, a cystic Bowman's space, and shrunken glomerular tuft, whereas the number of glomerular generations and total glomerular number did not differ [42]. The proportion of abnormal glomeruli ranged from 0.2 to 18%, suggesting that preterm birth may not adversely impact kidney development equally. Since premature baboons received antibiotics after birth (gentamicin and ampicillin), it is conceivable that the glomerular changes observed in this group resulted from antibiotic-induced nephrotoxicity. Because kidney development in nonprimate experimental models differs from that of primates (e.g., nephrogenesis continues postnatally in the mouse or rat), observations made in this study are more relevant for human disease. Indeed, preterm infants also have a higher percentage of enlarged glomeruli compared with stillborn gestational controls [41]. However, there was no difference in kidney or body weight at autopsy between SGA and AGA infants. This may be due to catch-up growth of the kidney postnatally after preterm birth in SGA neonates or to an insufficient power of the study due to a small number of preterm SGA infants (6 of 28). In addition, the width of the nephrogenic zone was lower in preterm than gestational control infants, implying a decreased capacity to form new nephrons in extrauterine environment [41]. Stillborn SGA infants or liveborn SGA infants who died within a year of birth had fewer nephrons than control stillborn AGA infants or control AGA neonates who died within a year of birth, respectively [22]. These findings suggest that the presence of IUGR might further reduce nephron number. Experimental study in mice demonstrated that premature delivery at 1-2 days prior to term birth caused a 20% decrease in glomerular number and resulted in elevated blood pressure, a lower measured GFR, and higher urine albumin/creatinine ratio at 5 weeks of age compared to full-term controls [35]. Since these effects of prematurity cannot be explained by unfavorable intrauterine conditions, it is conceivable that transition to extrauterine environment leads to premature termination of nephrogenesis by depriving the developing kidney from maternal factors essential for kidney development. Some observational studies in humans seem to agree with this possibility [39, 41, 45]. Collectively, these observations suggest that: (1) preterm kidneys may have fewer functional nephrons, thereby increasing vulnerability to impaired renal function in both the early postnatal period and later in life and (2) compensatory mechanisms in surviving preterm infants include glomerular hypertrophy that could lead to hyperfiltration. Overall, association of prematurity alone without IUGR with later kidney dysfunction or hypertension in children is subtle and requires further investigations.

### 3.4. Can Programmed Hypertension Be Dissociated from Reduced Nephron Number?

Even though the combination of Barker and Brenner hypotheses offers an explanation for the association of a reduced nephron number with hypertension and renal disease, no definitive proof has been found that low nephron endowment per se causes increased risk for hypertension or renal injury. Moreover, some animal studies demonstrate that reduced nephron number per se does not appear to mediate development of later hypertension. In a study by Hoppe et al., nephron number as well as conscious mean arterial pressure was reduced on postnatal day 135 in rats born to mothers fed a low-protein isocaloric diet throughout gestation and postnatally compared to rats fed a normal protein diet [16]. The authors proposed that the apparent blood pressure-lowering effect of life-long dietary protein restriction may be due to effects mediated during the postnatal period. Although the nature of postnatal factors that may be responsible for the observed effect on blood pressure remains to be elucidated, one potential mechanism may involve a shift in the pressure natriuresis relation towards lower mean arterial pressure. Because nephrogenesis continues postnatally in the rat, Wlodek et al. [52] hypothesized that nephron endowment and blood pressure of male offsprings born to placentally restricted mothers can be modulated by altering the postnatal (lactational) environment by cross-fostering. Uteroplacental insufficiency, created by ligation of uterine arteries on gestational day 18 (of 21), resulted in impaired mammary gland function, a twofold reduction in litter size, neutropenia, and adult hypertension. Cross-fostering of these newborn pups onto mothers with normal lactation prevented the nephron deficit and hypertension [52]. In contrast, pups born to mothers without ligation of uterine arteries where litter size was reduced at birth to match the spontaneously low litter size observed in uteroplacental insufficiency group and then cross-fostered at birth onto mothers subjected to ligation of uterine arteries had no nephron deficit but developed hypertension. The authors concluded that: (1) prenatally induced nephron deficit can be corrected by providing normal lactation postnatally, and (2) programmed hypertension can be dissociated from reduced nephron number in uteroplacental insufficiency model. These findings do not prove a causal relationship between restoration of nephron endowment by improved postnatal lactation and prevention of hypertension. It is conceivable that optimized postnatal nutrition affected development of the cardiovascular system or other factors (e.g., vasoactive factors) that regulate blood pressure to prevent the onset of hypertension independent of the effect on nephrogenesis. Given that 80% of nephrons in the rat kidney form in the first 10 days after birth, it is equally possible that the nephron deficit in offsprings of placentally restricted mothers was generated during embryonic or early postnatal period [53]. New studies examining nephron number at birth and at specific postnatal time points are required to determine whether nutritional rescue prevented nephron deficit or overcame a preexisting deficit by “accelerating” postnatal nephrogenesis. Together, these observations demonstrate that the postnatal environment is also important in determining the outcomes of developmental programming and that reduced nephron number per se does not appear to mediate all programmed hypertension. In addition, there are significant differences between reduced nephron number early in life and loss of nephrons later in life (e.g., kidney donation as an adult) in terms of later renal disease or hypertension. Studies in rats showed that unilateral nephrectomy during the later stages of kidney development (when nephrogenesis still continues) results in a higher GFR when compared to nephrectomy during early adulthood (after completion of nephrogenesis) [54]. This presumably reflects much more vigorous compensatory mechanisms in the developing organism.

### 3.5. Impact of Gestational Environmental Factors on Renal and Cardiovascular Outcomes

A growing body of evidence indicates that developmental programming of blood pressure and renal dysfunction is linked to maternal health conditions (eg., CKD and gestational diabetes), undernutrition, dietary deficiencies, and exposure to certain substances during gestation (Table 2). Uteroplacental insufficiency, one of the most common causes of IUGR, occurs in 7–10% of pregnancies and is usually due to poor maternal health, maternal cigarette smoking, or maternal undernutrition [59–61]. Approximately 25% of pregnant women smoke throughout their pregnancy, and smoking is one of the most modifiable risk factor for IUGR in developed economies [59–61]. Exposure to cigarette smoking *in utero* correlates with an increase in BP in the offspring in adulthood [62, 63]. Nicotine, the main component in cigarettes, induces vasoconstriction and decreases placental blood as well as oxygen delivery to the fetus resulting in aberrant fetal vascular development [93, 94]. In addition, maternal smoking stimulates production of the vasoconstrictor and thromboxane A2 [93]. Renal renin expression was noted to be decreased in animal models of uteroplacental insufficiency, leading to decreased RAS activity during nephrogenesis [90]. Additional factors shown to be associated with decreased nephron endowment and hypertension in children include maternal use of cocaine or alcohol during gestation [64]. Ethanol may act synergistically with estradiol in the development of renal abnormalities such as hydronephrosis, a congenital anomaly commonly seen in fetal alcohol syndrome [65, 66]. Another mechanism implicated in renal dysfunction associated with prenatal ethanol exposure includes an increase in cell death in the region of the developing nephric duct and in Na-K-ATPase activity in the renal cortex [67]. Use of indomethacin for tocolysis is associated with fetal renal impairment with oliguria and oligohydramnios [95–97]. Studies have shown that exposure to NSAIDS *in utero* resulted in decreased GFR secondary to reduction in renal blood flow, abnormal glomerular, and tubular development [80]. The observations that a number of perinatal insults such as maternal malnutrition, exposure to medications or toxins during pregnancy, or uteroplacental insufficiency have been associated with later hypertension or CKD suggest that the programming is not specific to a single-maternal factor. 

### 3.6. Mechanisms That Mediate the Impact of Gestational Environmental Factors on Renal and Cardiovascular Outcomes

Although our understanding of the underlying mechanisms implicated in programming of hypertension and CKD is far from complete, experimental models have provided certain mechanistic information. Animal models evaluating the effects of a low-protein diet during pregnancy have shown that offsprings with reduced number of glomeruli who develop hypertension in adulthood have altered expression of genes controlling metanephric organogenesis [35, 55, 56, 77, 83, 98–102]. The critical role of mutations in genes that direct metanephric organogenesis in developmental programming of renal dysfunction and blood pressure is supported by observations that polymorphisms in paired box 2 (*Pax-2*) or *Ret*, genes shown to be critical for normal kidney development in animal models, are associated with reduced renal volume in humans and may therefore associate with a reduced number of nephrons [103, 104]. Exposure to maternal low-protein diet *in utero* in the rat causes hypertension associated with microvascular rarefaction (reduced density of arterioles and capillaries) and decreased angiogenesis [105, 106]. Notably, microvascular rarefaction is associated with hypertension [107]. Another mechanism by which maternal low-protein diet may cause renal hypoplasia in an offspring is by increasing concentration of glucocorticoids *via* downregulation of the placental steroid-metabolizing enzyme, 11 *β* -hydroxysteroid dehydrogenase type 2 (11*β* HSD2) [56]. Decreased 11*β* HSD2 activity increases endogenous cortisol levels and leads to an increased plasma volume secondary to enhanced renal sodium retention and eventually to salt-sensitive hypertension [68]. The RAS plays an important role in fetal growth restriction and the development of hypertension in response to maternal low-protein diet. Decreased nephron endowment and glomerular hypertrophy are accompanied by suppression of the newborn intrarenal RAS, system essential for normal kidney development [99–102]. In addition, maternal low-protein diet increases the risk of salt-sensitive hypertension [57]. Observed salt sensitivity in an offspring may be due to increased expression of Na-K-2Cl (NKCC2) cotransporter in the thick ascending limb of the loop of Henle or decreased activity of the Na-K-ATPase in the inner medullary collecting duct [108, 109]. Administration of ouabain, a Na-K-ATPase ligand, to these rats abolished apoptosis and increased cell proliferation in the metanephric blastema [110, 111]. Oxidative stress and subsequent inflammation may be another important factor in programming hypertension. In this regard, maternal low-protein diet in the rat elicits oxidative stress and inflammation in the offspring [112]. It is postulated that low-protein diet results in a relative deficiency of nitric oxide (NO), a powerful vasodilator, and that supplementation with L-arginine, a NO donor, may be protective [58, 113–115]. Supplementation of lipid peroxidation inhibitor in pregnant rats fed low-protein diet prevents an elevation in blood pressure and improves vasodilatation and microvascular rarefaction in the offspring [116]. 

The importance of glucocorticoids in nephron endowment and programming of hypertension is demonstrated by the findings that administration of exogenous glucocorticoids to pregnant rats leads to reduced nephron number, possibly *via* downregulation of UB branching morphogenesis genes and results in hypertension in adult life [68, 69, 78, 117, 118]. Maternal administration of glucocorticoids during the first trimester in sheep leads to normal birth weight with subsequent development of high blood pressure in both sexes [117, 118]. Observed programming of high blood pressure may be due to aberrant UB branching morphogenesis and decreased glomerulogenesis that may be secondary to alterations in the intrarenal RAS [117]. Supplementing maternal diet with omega-3 fatty acid prevents dexamethasone-induced hypertension, hyperleptinemia, and upregulation of renal Na-K-ATPase activity in the offspring, thus providing an opportunity for potential therapeutic interventions [79]. Given that exposure to dexamethasone in animal models of IUGR stimulates activity of Na^+^-H^+^ exchanger in the proximal tubules; enhanced tubular reabsorption of sodium may play a role in developmental programming of hypertension [79]. An important role for estrogen in programming of blood pressure is evident from the observations that estrogen replacement in ovariectomized animals normalizes blood pressure in animal model of IUGR [119]. Administration of angiotensin (Ang) II to IUGR rats potentiates the observed increase in BP, suggesting that the RAS is important in the pathogenesis of hypertension in this model. IUGR rats of both sexes have decreased levels of vascular endothelial growth factor (VEGF), a growth factor critical for normal nephron endowment [91].

Increased maternal salt intake can result in renal structural and functional changes similar to those produced by gestational protein restriction [70, 71]. Both excessively high and low maternal sodium intakes during pregnancy in the rat cause aberrant expression of genes critical for normal metanephric organogenesis and reduce the final number of glomeruli in the offspring, predisposing to hypertension later in life [76, 120]. Occurrence of renal hypodysplasia caused by high maternal salt intake during gestation in bradykinin *B2 receptor*-deficient mice provides proof of the principle that environmental factors may act in concert with single-gene mutations to cause aberrant kidney development [121]. 

Experimental and observational studies suggest that premature birth can also adversely affect both vascular development and function [122]. Reduced density of arterioles and capillaries is associated with hypertension [107]. The importance of vascularization in programming of later hypertension is supported by the findings that reduced retinal vascularization observed in the preterm infants is associated with an increased risk for developing hypertension [123]. Neonates of mothers with preeclampsia (gestational hypertension) have increased aortic intima-media thickness and elevated serum triglyceride levels [124]. LBW is associated with an increased arterial wall stiffness in adolescents and adults [47, 125]. Impaired endothelial-dependent arterial relaxation, an early marker for the development of hypertension, may persist to adult life in LBW infants [23, 24]. These events may result in an increase in cardiovascular risk later in life. Given that deficiency of elastin in the arterial wall is the major determinant of arterial wall stiffness and that elastin synthesis in the aorta decreases rapidly after birth, decreased elastin content may be a likely cause of stiffer arteries and an increased risk of hypertension and cardiovascular disease in later life [125]. 

Oxidative stress has been implicated in developmental programming of hypertension. The fetus is hypoxic under physiologic conditions compared with the adult. Blood oxygen content increases abruptly after birth, leading to the generation of oxygen-free radicals [126]. Premature infants have immature antioxidant systems to respond to oxidative stress occurring during the transition to extrauterine life or need for oxygen therapy because of lung immaturity [127]. Gestational maternal protein restriction in rats results in impairment of antioxidant defenses as well as shorter aortic telomere length characteristic of vascular atherosclerosis, thus providing a possible mechanistic link between developmental insults and cardiovascular disease [128]. Our understanding of the mechanisms underlying possible long-term consequences of aberrant vascular structure and function, oxidative stress, and developmental kidney morphology in susceptible individuals is inadequate. Together, studies in animal models indicate that a number of specific perturbations are associated with aberrant nephron endowment in response to gestational environmental factors and subsequent risk of hypertension or CKD. These alterations include altered expression of genes controlling metanephric organogenesis, aberrant cell proliferation, survival and differentiation, fetal exposure to higher glucocorticoid levels, renal oxidative stress and inflammation, and increased expression of renal transporters promoting salt retention. 

### 3.7. Impact of Postnatal Environmental Factors on Renal and Cardiovascular Outcomes

Emerging data demonstrate that the early postnatal period is an additional developmental phase that is also susceptible to developmental programming. Rapid postnatal weight gain in LBW neonates is associated with childhood hypertension [129]. LBW infants who gained weight rapidly during childhood (1 to 5 years) had the highest blood pressure [130]. In a mice model, feeding high-fat diet after caloric restriction is associated with lipid accumulation and insulin resistance [131]. Children with extrauterine growth restriction (EUGR) (AGA with weight or height below the 10th percentile at discharge from NICU) have decreased measured GFR compared with normotrophic controls at mean age of 7.6 ± 1.3 years, suggesting that EUGR is a risk factor for subsequent impairment of renal function in premature neonates [48]. Premature neonates are also at risk of iatrogenic injury from administration of steroids, NSAIDS/COX-2 inhibitors or nephrotoxic antibiotics postnatally [81, 82]. Exposure to COX-2 inhibitors during nephrogenesis results in low nephron endowment and hypertension at birth that persists through adulthood [84]. Administration of COX-2 inhibitor to rats with altered renal development induces a greater renal vasoconstriction [84, 85]. Use of glucocorticoids is a standard of care to accelerate lung development in preterm births. Recent studies demonstrate that premature Caucasian individuals carrying a N363S variant of the glucocorticoid receptor (GR), described to be associated with higher sensitivity to glucocorticoids, develop abdominal adiposity and insulin resistance when exposed to antenatal glucocorticoids [86, 87]. In contrast, carriers of the GR ER22/23 K variant, noted to be associated with lower sensitivity to glucocorticoids, are protected against insulin resistance after preterm birth [86, 87]. Limitations of the study by Finken et al. include a small number of subjects heterozygous for N363S variant (*n* = 4) and nonrandomized assignment of glucocorticoid therapy in an observational study. Basic *in vitro* and *ex vivo* studies demonstrate that different sensitivity to glucocorticoids is due to alterations in transactivating capacity of the GR [132]. These findings support the possibility that glucocorticoid sensitivity-modulated polymorphisms of GR may be important in linking glucocorticoid excess *in utero* to cardiovascular and metabolic diseases in adulthood. Preterm and SGA (birth weight less than 10th percentile for gestational age) neonates usually undergo a period of accelerated postnatal growth that enhances the risk of obesity and elevated blood pressure later in life [133]. In turn, for each 1 kg increase in birth weight, the odds for developing elevated systolic and diastolic blood pressure at 7 years of age are increased by 2.19 and 1.82, respectively [134]. One potential mechanism that may account for susceptibility to high blood pressure in obese neonates may involve increased production of angiotensinogen (AGT), a source for angiotensin (Ang) II [135, 136]. It also appears that renal developmental programming of hypertension may be sex dependent [89]. Testosterone tends to enhance the vasopressor arm while estrogen enhances the vasodepressor arm. Only male rats develop lower nephron numbers, higher blood pressure, and lower GFR and proteinuria when exposed to Ang II blockade or COX-2 inhibition during nephrogenesis [82, 89]. Early postnatal overfeeding induces early chronic renal dysfunction in adult male, but not female rats [137]. In summary, early postnatal factors may also program an increase in later cardiovascular risk in both LBW and premature infants. Therefore, it will be important to elucidate how to optimize care in the setting of NICU to achieve optimal kidney development in LBW or premature neonates after birth. 

### 3.8. Role of Epigenetic Factors in Kidney Development and Programming of Renal Disease and Hypertension

Epigenetic modifications provide one potential mechanism for how environmental influences in early life cause long-term changes in chronic disease susceptibility. The major players in epigenetic mechanisms of gene expression regulation are DNA or chromatin protein methylation, acetylation, and chromatin remodeling. Posttranslational modifications of histones such as histone acetylation and methylation of cytosine bases adjacent to guanines (CpG dinucleotides) may affect chromatin function and alter gene expression in the absence of changes in DNA sequence [138, 139]. It has been shown that a maternal low-protein diet or tobacco use is associated with reduced global methylation in the liver of the offspring in the rat or in the human placenta, a metabolic and endocrine organ that may be considered an “imprint” of fetal exposure *in utero* [140, 141]. For example, maternal smoking deregulates placental methylation of CpG dinucleotides, which correlates with alterations in the expression of gene pathways involved in regulation of cell death, morphology, signaling, and metabolism [141]. Since these gene pathways are likely of biological and clinical significance, observed alterations in gene methylation have the potential to impact the health of the offspring. 

Relevant to the regulation of normal and abnormal kidney development, a recent study demonstrated a link between Pax2, a transcription factor critical for renal morphogenesis, and chromatin methylation [142]. *Pax 2* gene encodes DNA-binding protein that can specify the intermediate mesoderm, a type of embryonic tissue that will subsequently generate the urogenital tract. The ubiquitous nuclear protein PTIP is an essential component of a histone H3 lysine 4 (H3 K4) methyltransferase complex that maintains active chromatin domains by H3 K4 methylation. Pax2 protein promotes assembly of an H3 K4 methyltransferase complex through PTIP, thereby linking DNA-binding regulators of kidney development to epigenetic imprinting [142]. Targeted deletion of *PTIP* in glomerular podocytes in mice led to altered expression of select genes whose function may be essential for podocyte foot process patterning, progressive proteinuria, and podocyte ultrastructural defects similar to chronic glomerular disease. These data demonstrate that alterations or mutations in an epigenetic regulatory pathway can alter the phenotypes of differentiated kidney cells and lead to a chronic disease state [143].

Activation of p53, a tumor suppressor protein, results in cell cycle arrest and apoptosis. Notably, tight regulation of p53 activity is an absolute requirement for normal kidney development [144]. Altered methylation of the *p53* gene has been observed in the full-term IUGR rat kidney [92]. Specifically, IUGR increases p53 and Bax (proapoptotic gene) and decreases Bcl-2 (anti-apoptotic gene) mRNA levels, leading to enhanced renal apoptosis and reduced glomerular number. These changes are accompanied by decreased CpG methylation of the renal *p53 *promoter. Thus, altered methylation of p53 may represent a mechanism that contributes to the fetal origins of adult kidney disease. An important role for such an epigenetic mechanism as histone acetylation in programming of kidney disease is supported by the observation that treatment of embryonic kidneys with histone deacetylase inhibitors (HDACis) impairs the ureteric bud branching morphogenesis program and provokes renal growth arrest and apoptosis [145]. In addition to DNA or chromatin protein methylation or acetylation, gene expression may also be regulated at the posttranscriptional level by noncoding microRNAs (miRNAs). miRNAs are small endogenous RNA molecules about 22 nucleotides in length. Dicer is an enzyme that cleaves precursor miRNAs into the mature miRNAs. Silencing of target gene expression by miRNAs occurs by binding of the mature miRNA to the target mRNA and preventing its translation or inducing its degradation [146]. The essential role for miRNAs in kidney development is evident from the observation that targeted genetic inactivation of *Dicer* in the UB in mice results in hydronephrosis and renal cysts [147]. These anomalies are most likely due to loss of mature miRNAs in the ureteric bud and its derivatives (renal pelvis, ureter, and collecting ducts), leading to deregulated expression of genes that affect renal collecting system development.

Whereas epigenetic dysregulation is increasingly implicated in the regulation of normal kidney development, the role of epigenetics in prenatal programming of such complex diseases as hypertension remains largely uncharacterized. Collecting duct epithelial sodium channel (ENaC) is critical in Na^+^ reabsorption in the distal tubule and hence the regulation of extracellular fluid volume and blood pressure [148]. Activating *ENaC* mutations cause Liddle's syndrome, a hereditary disease characterized by hypertension [149]. Aldosterone, a major regulator of epithelial Na^+^ absorption, activates ENaC to increase extracellular fluid volume and blood pressure. Recent studies reveal a novel role for epigenetic mechanisms in mediating aldosterone-induced control of *ENaC* gene expression. Aldosterone-induced H3 K79 hypomethylation at specific subregions of *ENaC alpha* promoter is associated with derepression of the *ENaC alpha* promoter in murine inner medullary collecting ducts cells [150]. It is conceivable that hypomethylation of *ENaC alpha *may lead to enhanced renal sodium reabsorption and thus potentially elevated blood pressure in humans. 

Promoter of a gene encoding for another solute carrier, Na^+^-K^+^-2Cl^−^ cotransporter 1 (*NKCC1*), normally expressed in vascular smooth muscle cells, is also subject to epigenetic regulation during postnatal development of hypertension in spontaneously hypertensive rat (SHR) [151]. Notably, NKCC1 is implicated in the maintenance of vascular tone *in vivo* since *NKCC1*-knockout mice have lower blood pressure [152]. In SHR, *NKCC1* promoter is hypomethylated, and expression of NKCC1 is increased after development of hypertension compared to control Wistar-Kyoto rats (WKY) [151]. Thus, hypomethylation of *NKCC1* plays an important role in the upregulation of NKCC1 during development of spontaneous hypertension, and increased NKCC1 expression in the vasculature may underlie elevated blood pressure [151]. These data suggest a link between epigenetic modification of genes and the resultant alteration in the expression of genes in pathways associated with a range of physiologic processes. 

**Figure 1:**
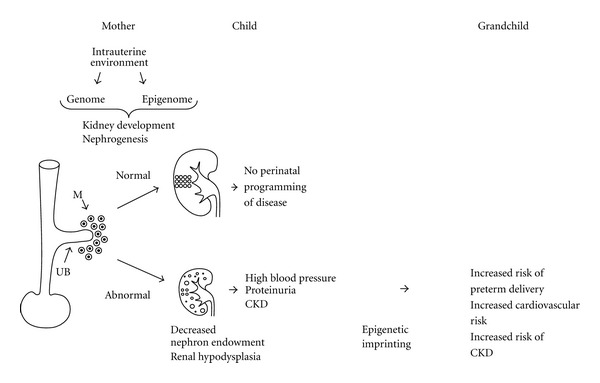
Schematic representation of the proposed impact of adverse intrauterine environment on developmental programming of hypertension and chronic kidney disease (CKD). Maternally mediated environmental modulation of renal gene expression in the offspring leads to developmentally induced deviations from the optimal nephron number. A relative deficiency in the number of nephrons is thought to create an increased risk of CKD, hypertension, and cardiovascular morbidity in later life. Epigenetic modifications not only change target gene expression and program the phenotype of the developing fetus, but also account for transgenerational inheritance of programmed phenotype *via* permanent epigenetic imprinting. UB: ureteric bud and M: metanephric mesenchyme.

Environmental influences during an individual's early life are not the sole cause of long-term changes in chronic disease susceptibility. Emerging data suggest that integration of signals from an individual's mother's lifetime nutritional or health experience contributes to intergenerational transfer of environmental information. For example, offsprings of LBW or preterm mothers are more likely to be born with LBW or preterm, indicating transgenerational effect for LBW or preterm birth [153]. Epigenetic imprinting, alteration of gene expression based on their methylation status, is likely to play a role in transmitting epigenetic information from previous generations [154] (Figure 1). 

## 4. Implications of the State of Current Knowledge Regarding the Role of the Kidney in Developmental Origins of Disease

Preterm birth and LBW are risk factors for the development of kidney disease and elevated blood pressure in later life. Most studies seem to demonstrate that the association between an adverse intrauterine environment and renal disease and hypertension in later life appears to be mediated, at least in part, by impaired kidney development and nephron endowment. Despite recent advances in elucidation of the cellular and molecular mechanisms linking intrauterine environment to kidney organogenesis and developmental origins of disease, including hypertension and CKD, our understanding of its cause in an individual patient is still too limited. Common diseases like hypertension are assumed to be multifactorial and occur as a result of a combination of epigenetic and environmental factors affecting genetically susceptible individual. The best available surrogate markers for low nephron number in children include LBW, IUGR, short stature, and reduced kidney volume on ultrasound and glomerulomegaly on kidney biopsy. Because morbidity in perinatal programming may not manifest until later in life, all patients who are at risk (eg., LBW and IUGR neonates) should be closely followed throughout life. Medical monitoring of these neonates should include avoidance of nephrotoxic medications, optimization of nutrition, growth, blood pressure, renal function, monitoring for proteinuria, obesity counseling, and urinary tract imaging when indicated. Gestational interventions should include optimization of prenatal care and maternal nutrition, avoidance of medications that may impair fetal growth or normal kidney development (e.g., RAS blockers), maternal smoking, and alcohol use. Introduction of more sensitive array-based methods and recently developed epigenomic technologies, which allow screening for multiple gene mutations and detection of epigenetic modifications, may unravel a complex network of molecular interactions. This will help to determine and predict the occurrence and consequences of impaired nephrogenesis, CKD, and hypertension. Improved understanding of epigenetic and other mechanisms of developmental programming of CKD and hypertension will facilitate development of novel interventional strategies targeted at prevention of these diseases in later life.

## Figures and Tables

**Table 1:** Effect of prematurity alone or prematurity with IUGR on postnatal kidney growth, morphology, and programming of renal dysfunction and blood pressure.

Author (reference)	Country	Study design	Summary of reported findings
Rodríguezet al. [39]	USA	An autopsy study of 56 extremely premature infants (*n* = 42 AGA, and *n* = 14 SGA)	Decreased RGC in premature AGA versus term Increased mesangial tuft area and Bowman's capsule area in preterm surviving >40 days without RF versus term or preterm surviving <40 days with or without RF
Keijzer-Veenet al. [40]	Netherlands	Determination of renal size at 20 years of age by ultrasonography in 81 individuals born preterm AGA, (*n* = 29), SGA (*n* = 22), or term (*n* = 30)	Decreased kidney size in both preterm AGA and SGA versus term. No difference in kidney size between AGA and SGA preterm
Drougia et al. [18]	Greece	Determination of kidney length at 2 years of chronologic age in 466 children (*n* = 223 AGA and *n* = 243 SGA)	Decreased kidney length in preterm SGA (<36 weeks of GA) versus preterm AGA
Schmidt et al. [20]	Denmark	Determination of kidney volume by ultrasonography at birth and at 18 months of age in preterm or term SGA (*n* = 178) versus term AGA (*n* = 717)	Reduced kidney volume at birth and 18 month of age in premature AGA versus term AGA and in preterm SGA versus preterm AGA.
Sutherland et al. [41]	Australia	Determination of kidney morphology on autopsy in 28 preterm neonates at 2–68 days after birth and 32 stillborn gestational controls	Higher percentage of enlarged glomeruli in preterm versus controls, no difference in kidney weight in preterm SGA versus preterm AGA
Hinchliffe et al. [22]	UK	Determination of total glomerular number and volume in stillborn AGA and SGA neonates, in liveborn AGA, and SGA infants who died within 1 year of birth	Decreased glomerular number in SGA versus AGA in both stillborn and those who died within 1 year after birth, no difference in glomerular volume
Gubhaju et al. [42]	Australia	Determination of kidney and glomerular size, glomerular density, glomerular morphology and number, and number of glomerular generations in preterm baboons studied after 21 days of extrauterine life versus GA-matched controls	Larger kidneys, decreased glomerular density, enlarged glomeruli, shrunken glomerular tuft, and cystic Bowman's space in preterm versus controls No difference in total number of glomeruli or number of glomerular generations
Stelloh et al. [35]	USA	Determination of the effect of preterm delivery at 1-2 days prior to term birth in mice on glomerular number, blood pressure, measured GFR, and urine albumin/creatinine ratio at 5 weeks of age	A 20% decrease in glomerular number, increased blood pressure, lower GFR and higher urine albumin/creatinine ratio in preterm versus term
Keijzer-Veen et al. [43]	Netherlands	Determination of blood pressure at 19 years of age in 422 individuals with GA < 32 weeks and in 174 individuals with GA > 32 weeks and birth weight <1500 g	Increased prevalence of elevated blood pressure in GA < 32 weeks versus GA > 32 weeks not related with IUGR, increased postnatal weight gain and weight at age of 19 affected the risk for hypertension
Singhal et al. [44]	UK	Determination of blood pressure and flow-mediated endothelial-dependent vasodilation (EDV) in preterm SGA (*n* = 72), preterm AGA (*n* = 144), and term AGA (*n* = 61) individuals at age 13–16 years	No difference in blood pressure among all groups and reduced EDV in preterm SGA versus preterm or term AGA
Leon et al. [45]	Sweden	Record linkage study of 165;136 men studied at mean age of 18 years	Inverse association of blood pressure with GA alone or with GA adjusted for birth weight (SGA) and increased inverse association of blood pressure in SGA versus AGA
Kistner et al. [46]	Sweden	Determination of systolic ambulatory blood pressure (ABP) at a mean age of 26 ± 2 years in woman born term SGA (*n* = 18), with term AGA (*n* = 17), and preterm AGA (*n* = 14)	Higher systolic ABP in preterm AGA versus term AGA or term SGA, no difference in term SGA versus term AGA
Cheung et al. [47]	China	Determination of mean blood pressure and brachioradial artery pulse wave velocity (PWV) in ex-preterm SGA (*n* = 15), preterm AGA (*n* = 36), and term AGA (*n* = 35) children at 8 ± 1.7 years of age	Higher mean blood pressure and PWV in preterm SGA versus preterm or term AGA
Keijzer-Veen et al. [31]	Netherlands	Determination of GFR and urine albumin/creatinine ratio at 19 years of age in individuals with GA < 32 weeks SGA (*n* = 215) or AGA (*n* = 207)	Decreased GFR and increased prevalence of high albumin/creatinine ratio in preterm SGA versus AGA
Bacchetta et al. [48]	France	Single-center prospective cohort study Determination of GFR measured by inulin clearance at mean age of 7.6 ± 1.3 years in preterm SGA (*n* = 23), AGA (*n* = 11), and preterm with EUGR (*n* = 16)	Lower GFR in SGA versus AGA and in EUGR versus AGA, no difference in urine albumin/creatinine ratio or in blood pressure among the groups

RGC: radial glomerular count, RF: renal failure, GA: gestational age, SGA: small for GA, AGA: appropriate for GA, GFR: glomerular filtration rate, EUGR: extrauterine growth retardation.

**Table 2:** Effect of environmental factors on kidney development and programming of renal dysfunction and blood pressure.

Factors	Phenotype	References
Maternal low-protein diet	LBW, decreased nephron number, and salt-sensitive hypertension	[16, 38, 55–58]
Maternal cigarette smoking	Hypertension	[59–63]
Alcohol use	Decreased nephron number	[64–67]
Steroids	Decreased nephron number and hypertension	[68–71]
Vitamin A deficiency	Rat-renal hypoplasia	[72]
Iron deficiency	Rat-decreased birth weight and hypertension Rat-decreased nephron number and hypertension	[73, 74]
High-salt diet	Rat-hypertension in an offspring, children-increased responsiveness of blood pressure to changes in dietary salt intake	[75, 76]
Glucocorticoid exposure	Decreased GFR and reduced number of nephrons hypertension	[56, 68, 77–79]
NSAIDs	Abnormal glomerular and tubular development	[80–82]
ACEi/ARBs	Renal tubular dysgenesis and hypotension	[83]
COX-2 exposure	Decreased nephron number and hypertension	[84, 85]
GR^N363S^	Obesity and increased insulin resistance	[86, 87]
GR^ER22/23K^	Protect against insulin resistance	[88]
Testosterone	Decreased nephron number and proteinuria, hypertension	[82, 89]
Uteroplacental insufficiency	Renal hypoplasia	[36, 52, 90–92]

LBW: low birth weight, GFR: glomerular filtration rate, NSAIDS: nonsteroidal anti-inflammatory drugs, ACEi: angiotensin-converting enzyme inhibitors, ARBs: angiotensin receptor blockers, COX-2: cyclooxygenase-2, and GR: glucocorticoid receptor.
